# Comparison of metabolomic reconfiguration between Columbia and Landsberg ecotypes subjected to the combination of high salinity and increased irradiance

**DOI:** 10.1186/s12870-023-04404-7

**Published:** 2023-08-25

**Authors:** Clara Segarra-Medina, Lidia S. Pascual, Saleh Alseekh, Alisdair R. Fernie, José L. Rambla, Aurelio Gómez-Cadenas, Sara I. Zandalinas

**Affiliations:** 1 Departamento de Biología, Bioquímica Y Ciencias Naturales, Universitat Jaume I, 12071 Castelló de La Plana, Spain; 2 Max Planck Institute of Molecular Plant Physiology, Am Mühlenberg 1, 14476 Potsdam, Germany

**Keywords:** Arabidopsis ecotypes, Abiotic stress combination, Metabolomics, Salinity, Increased irradiance

## Abstract

**Background:**

Plants growing in the field are subjected to combinations of abiotic stresses. These conditions pose a devastating threat to crops, decreasing their yield and causing a negative economic impact on agricultural production. Metabolic responses play a key role in plant acclimation to stress and natural variation for these metabolic changes could be key for plant adaptation to fluctuating environmental conditions.

**Results:**

Here we studied the metabolomic response of two Arabidopsis ecotypes (Columbia-0 [Col] and Landsberg erecta-0 [Ler]), widely used as genetic background for Arabidopsis mutant collections, subjected to the combination of high salinity and increased irradiance. Our findings demonstrate that this stress combination results in a specific metabolic response, different than that of the individual stresses. Although both ecotypes displayed reduced growth and quantum yield of photosystem II, as well as increased foliar damage and malondialdehyde accumulation, different mechanisms to tolerate the stress combination were observed. These included a relocation of amino acids and sugars to act as potential osmoprotectants, and the accumulation of different stress-protective compounds such as polyamines or secondary metabolites.

**Conclusions:**

Our findings reflect an initial identification of metabolic pathways that differentially change under stress combination that could be considered in studies of stress combination of Arabidopsis mutants that include Col or Ler as genetic backgrounds.

**Supplementary Information:**

The online version contains supplementary material available at 10.1186/s12870-023-04404-7.

## Background

Plants growing in the field are naturally exposed to a wide variety of abiotic and biotic stresses that reduce their growth and development. Recently, climate change and global warming are increasing the frequency and intensity of these environmental constrains, leading to devastating losses in crop yield worldwide [1]. Among the different environmental conditions that negatively impact plants, high salinity is considered a major yield-limiting condition that poses an important threat to agriculture [2, 3]. Increased concentrations of salt in soils arise as a result of reduced quality of irrigation water [3] and cause a negative economic impact on agricultural crop production accounting for more than US $27.3 billion [4]. Plants subjected to high salinity accumulate different osmolytes to adjust osmotic disorders, as well as alter their physiology and metabolism in order to re-establish the cellular redox balance [3, 5, 6]. Previous reports have demonstrated both conserved and divergent metabolic responses to salt acclimation across different species including *Arabidopsis thaliana*, *Lotus japonicus* and *Oryza sativa*, and found that a shift in the balance between amino acids and organic acids could be a common metabolic adaptation of plants in response to salt-induced stress [7]. The effect of high salinity on plants could be exacerbated by the concurrent action of other harmful abiotic stresses such as increased irradiance. During increased irradiance intensities, irreversible damage to photosynthetic reaction centers can occur, leading to photoinhibition [8, 9]. Due to the importance of light for photosynthetic organisms, plants evolved a wide range of adaptation strategies to prevent the damaging effects of increased irradiance, including mechanisms to adjust the size of the antenna complexes and the accumulation of metabolites to scavenge the excess of reactive oxygen species (ROS) [10–12]. Therefore, plants deploy specific physiological, molecular and metabolic mechanisms to respond and acclimate to different fluctuating environmental conditions. In this sense, it has been widely reported that the combination of different abiotic stresses elicits, in general, specific responses compared to those shown under individual stresses (*e.g*., [13–16]). Among these responses, metabolic adjustments aimed at accomplishing a new state of homeostasis are key for plant acclimation to stress [17].

Plants can produce a vast number of different compounds classified as primary metabolites such as sugars, amino acids or polyamines, that are crucial for cell functions including nutrition, development and reproduction. In addition to primary metabolites, secondary metabolites (also known as specialized metabolites) such as phenolics, terpenes, and nitrogen-containing compounds, as well as plant hormones (including abscisic acid [ABA], jasmonic acid [JA] or salicylic acid [SA], among others) are essential for plants to respond to fluctuations in their environment [18]. These compounds act as potent regulators of growth and development as well as antioxidants, protect membranes and, overall, promote plant tolerance to stress [18–20].

Metabolomic studies of transgenic or mutated *Arabidopsis thaliana* compared to wild type plants in response to stress have expanded the insights into the role of different metabolites for plant acclimation to stress (*e.g.* [15, 16, 19, 21–26]*,*). Columbia-0 (Col) and Landsberg erecta-0 (Ler) are common ecotypes of *Arabidopsis thaliana* widely used in molecular and genetic studies as genetic backgrounds for the majority of Arabidopsis T-DNA insertion mutant collections [27]. Genomes of both Col and Ler plants are whole sequenced, showing differences at the Single Nucleotide Polymorphism (SNP) level as well as in large indels (numerous events of insertions or deletions; [28, 29]), explaining their phenotypic differences. For example, in contrast to Col, Ler shows round leaves with short petioles and top clustered flowers [30]. In addition, distinct metabolic alterations in Arabidopsis inflorescences between Col and Ler ecotypes have been also described [31].

It has been previously reported that both accessions trigger different strategies to respond to stress. For example, Ler plants produce more lateral root primordia and overall larger root system compared to that of Col in response to osmotic stress [32]. In response to drought stress, both ecotypes show different stress-adaptive mechanisms: whereas Ler plants exhibit an escape strategy accelerating flowering, Col plants display a drought tolerance strategy [33]. In comparison to Col, Ler accumulates higher amounts of AtAVP1 transcript that are correlated with higher salinity tolerance when growing in hydroponics medium [34]. Therefore, genetic and/or metabolomic variations among the different Arabidopsis ecotypes could identify new targets for crop improvement.

The aim of this article was to study phenotypic and metabolic differences between Col and Ler plants subjected to the combination of high salinity and increased irradiance. Our findings showed that the metabolic response of Arabidopsis plants to this stress combination is different than that of plants subjected to the individual stresses. We further identified differences and similarities in the accumulation of primary and secondary compounds between both Arabidopsis ecotypes subjected to stress combination that could be involved in different mechanisms to tolerate the combination of high salinity and increased irradiance.

## Results

### Growth and physiological responses of Arabidopsis plants subjected to the combination of high salinity and increased irradiance

**Fig. 1:**
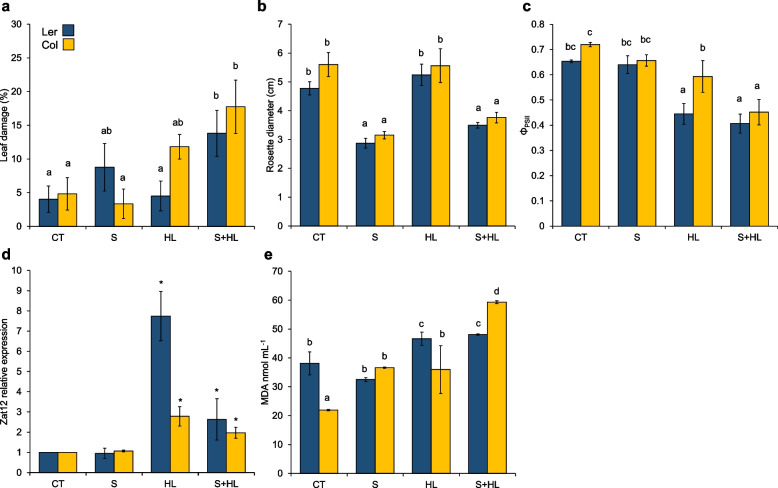
Growth, leaf damage, quantum yield of photosystem II, Zat12 relative expression and MDA accumulation of Col and Ler plants subjected to a combination of high salinity and increased irradiance. **a** Percentage of leaf damage in Col and Ler plants subjected to S, HL and S + HL. **b** Rosette diameter of Col and Ler plants subjected to S, HL and S + HL. **c** Quantum yield of PSII of Col and Ler plants subjected to S, HL and S + HL. **d** Relative expression of the transcriptional regulator Zat12 in Col and Ler plants subjected to S, HL and S + HL. **e** MDA accumulation in Col and Ler plants subjected to S, HL and S + HL. Error bars represent SE. Different letters denote statistical significance at *P* < 0.05. Asterisks denote statistical significance at *P* < 0.05 with respect to control values. CT, control; HL, increased irradiance; MDA, malondialdehyde; PSII, photosystem II; S, high salinity; S + HL, a combination of high salinity and increased irradiance; Φ_PSII_, quantum yield of photosystem II

To study the growth, quantum yield of photosystem II and stress-related transcriptomic and biochemical responses (Zat12 relative expression and malondialdehyde [MDA] accumulation) of Arabidopsis plants in response to conditions of high salinity, increased irradiance and the combination of both factors (Fig. 1), we subjected Col and Ler plants to 150 mM NaCl for 10 days (S), 650 μmol m^−2^ s^−1^ increased irradiance for 7 h (HL) or the combination of 150 mM NaCl for 10 days and 650 μmol m^−2^ s^−1^ increased irradiance for 7 h (S + HL; Fig. S1). Individual stresses did not significantly alter the percentage of damaged leaves in both ecotypes compared to control plants. The percentage of damaged leaves, however, significantly increased in Col (around 19%) and in Ler plants (around 14%) in response to S + HL, demonstrating that the stress combination had a higher impact on plants of both ecotypes compared to individual stress conditions (Fig. 1a). In addition, stress imposition had a similar impact on Col and Ler growth, and the effect of high salinity (10 days of stress) prevailed over the effects of increased irradiance (7 h of stress) on plant growth. In this sense, rosette diameter decreased in response to S or S + HL in both ecotypes, whereas HL stress did not alter plant growth compared to control values (Fig. 1b). In contrast, photosystem II (PSII) performance in terms of quantum yield of PSII was significantly affected by the exposure to HL conditions applied individually or in combination with high salinity (HL and S + HL) with respect to control values in Col and Ler plants (Fig. 1c). HL-induced stress on plants was further evidenced by the induction of the ROS- and light-responsive transcript Zat12 [35–37], along with the MDA accumulation as a marker of oxidative stress [38] in both ecotypes subjected to increased irradiance treatments (HL and S + HL; Fig. 1d, e).

### Accumulation of primary metabolites in Arabidopsis plants subjected to the combination of high salinity and increased irradiance

**Fig. 2:**
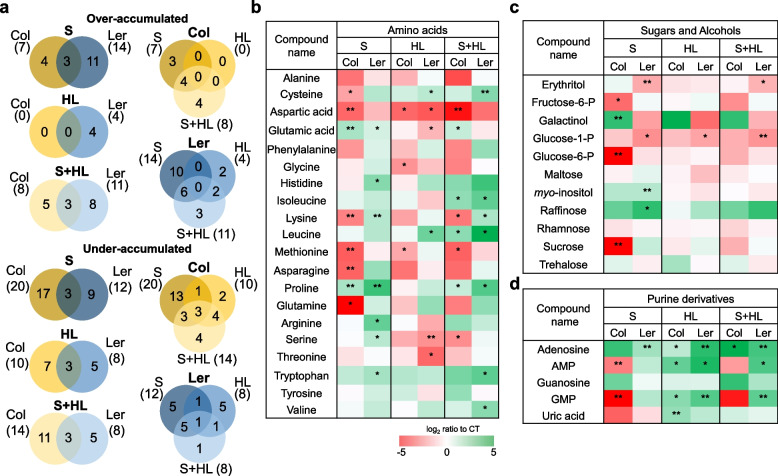
Differential accumulation of primary metabolites in Col and Ler plants subjected to a combination of high salinity and increased irradiance. **a** Venn diagrams depicting the overlap among over-accumulated (top) and under-accumulated (bottom) primary metabolites in Col and Ler plants subjected to S, HL and S + HL. A comparison of the metabolite changes between both ecotypes in each stress condition is shown on the left and a comparison of the metabolite changes among the different stress treatments (S, HL and S + HL) in each ecotype is shown on the right. **b**-**d** Levels of amino acids (**b**), sugars and alcohols (**c**), and purine derivatives (**d**) in Col and Ler plants subjected to S, HL and S + HL. Metabolite levels are expressed as log_2_ of ratio to control values and are shown as a color scale. Asterisks denote significant metabolite level (*P < 0.05; **P < 0.01) compared to control conditions. AMP, adenosine monophosphate; CT, control; GMP, guanosine monophosphate; HL, increased irradiance; S, high salinity; S + HL, a combination of high salinity and increased irradiance

**Fig. 3:**
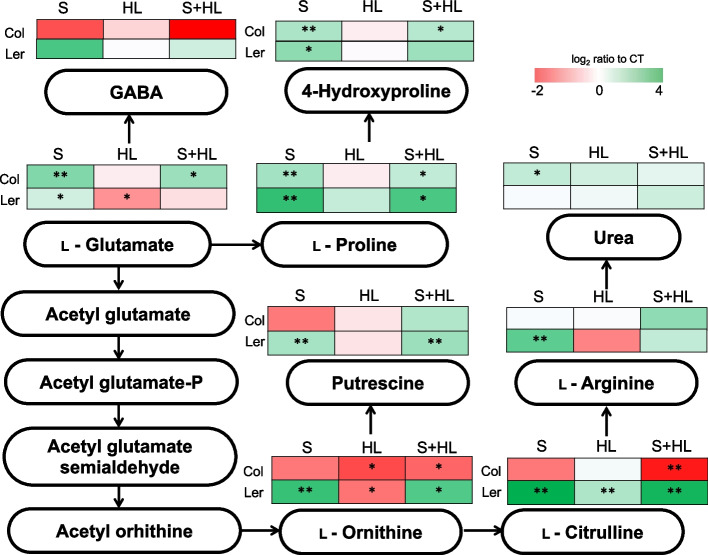
Glutamate metabolism in Col and Ler plants subjected to a combination of high salinity and increased irradiance. Level of metabolites involved in glutamate metabolism are shown as heat maps in Col and Ler plants subjected to S, HL and S + HL. Metabolite levels are expressed as log_2_ of ratio to control values and are shown as a color scale. Asterisks denote significant metabolite level (*P < 0.05; **P < 0.01) compared to control conditions. CT, control; GABA, γ-aminobutyric acid; HL, increased irradiance; S, high salinity; S + HL, a combination of high salinity and increased irradiance

To study differences and commonalities between both Arabidopsis wild type plants in the accumulation of different primary metabolites (*i.e.*, sugar, alcohols, amino acids, purine derivatives and polyamines) in response to S, HL and S + HL, a gas chromatography–mass spectrometric analysis of polar compounds was performed (Figs. 2 and 3; Tables S1, S2). Different accumulation patterns of primary metabolites between Col and Ler were observed in response to individual and combined stress (Fig. 2a), suggesting significant divergences between both ecotypes in reconfiguring primary metabolism in response to stress. In Col plants, S triggered the over- and under-accumulation of 7 and 20 primary metabolites, respectively, whereas in Ler plants, 14 and 12 primary metabolites were over- and under-accumulated, respectively. HL induced the over- and under-accumulation of 4 and 8 primary metabolites in Ler plants, respectively, whereas the level of 10 metabolites were attenuated with respect to CT in Col plants. S + HL increased the accumulation of 8 and 11 primary metabolites in Col and Ler plants, respectively, and decreased the level of 14 metabolites in Col plants and 8 metabolites in Ler plants (Fig. 2a; Table S1). Interestingly, 4 and 3 primary metabolites were found exclusively over-accumulated in Col and Ler plants, respectively, in response to S + HL. Similarly, levels of 4 and 1 primary metabolites decreased exclusively in response to S + HL in Col and Ler plants, respectively (Fig. 2a; Table S1).

In general, S differently altered amino acid content in Col and Ler plants (Fig. 2b; Table S1). Therefore, in Col plants, S induced a reduction in the levels of several amino acids, including cysteine, aspartic acid, lysine, methionine, asparagine and glutamine whereas it caused the accumulation of glutamic acid and proline levels. In Ler plants, however, contents of tryptophan, serine, arginine, proline, lysine, histidine and glutamic acid increased in response to S. HL induced a decrease in aspartic acid levels in both ecotypes, but differently altered the levels of other amino acids in Col and Ler plants. In this sense, whereas levels of glycine and methionine decreased in Col plants, cysteine and leucine values increased and glutamic acid and serine levels decreased in Ler plants. In response to S + HL, the accumulation of different branched-chain amino acids (isoleucine and leucine) as well as proline increased in both Col and Ler plants. Other amino acids that significantly accumulated under S + HL included valine, cysteine, lysine and tryptophan in Ler, and glutamic acid and arginine in Col. Whereas in Ler plants subjected to S + HL amino acids were always over-accumulated compared to CT values, in Col plants, levels of these metabolites decreased in response to S + HL, including aspartic acid, serine, lysine and methionine (Fig. 2b; Table S1).

Soluble sugar and sugar-derived alcohol levels differed depending on the stress treatment and ecotype (Fig. 2c; Table S1). Levels of glucose-1-P decreased in response to all stress treatments in Ler plants, while Col plants only showed decreased values of fructose-6-P and glucose-6-P in response to S. In addition, Ler plants subjected to S triggered reduced levels of erythritol and increased values of *myo*-inositol and raffinose compared to CT (Fig. 2c; Table S1).

Purine metabolism was differentially regulated in Col and Ler plants subjected to stress. Whereas S induced an accumulation of adenosine in Ler plants, this stress condition triggered a decrease in AMP and GMP levels in Col plants. HL, in addition, induced the over-accumulation of AMP, GMP and adenosine content in both ecotypes, whereas S + HL increased adenosine levels in both ecotypes, and AMP and GMP content only in Ler plants (Fig. 2d; Table S1).

Analysis of the glutamate metabolism and polyamine biosynthesis (Fig. 3; Table S2) revealed differences between Col and Ler in response to individual and combined stresses. In Ler plants subjected to S and S + HL, glutamate diverted into proline and ornithine accumulation. Ornithine, in turn, was converted into putrescine and citrulline under these stress conditions, showing these compounds increased levels with respect to CT in response to S and S + HL. In addition, Ler plants accumulated 4-hydroxyproline and arginine in response to S, whereas HL induced the over-accumulation of citrulline but a decrease in the levels of its precursor, ornithine (Fig. 3; Table S2). In contrast to Ler, in Col plants subjected to S and S + HL, glutamate only diverted into proline and its derivate 4-hydroxyproline, and the accumulation of ornithine and citrulline decreased in response to S + HL. Additionally, Col plants subjected to S triggered the over-accumulation of urea and HL-treated Col plants significantly repressed ornithine levels with respect to CT. In contrast to Col, Ler plants significantly accumulated putrescine in response to S and S + HL whereas no significant change in its levels was observed in Col in response to any stress treatment (Fig. 3; Table S2).

### Hormonal accumulation of Arabidopsis plants subjected to the combination of high salinity and increased irradiance

**Fig. 4:**
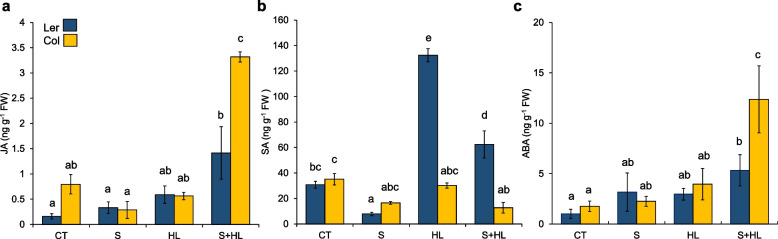
Hormonal levels in Col and Ler plants subjected to a combination of high salinity and increased irradiance. **a**-**c** JA (**a**), SA (**b**), and ABA (**c**) levels in Col and Ler plants subjected to S, HL and S + HL. Error bars represent SE. Different letters denote statistical significance at *P* < 0.05. ABA, abscisic acid; CT, control; HL, increased irradiance; JA, jasmonic acid; S, high salinity; SA, salicylic acid; S + HL, a combination of high salinity and increased irradiance

To study differences in hormone profiles between Col and Ler plants, jasmonic acid (JA), salicylic acid (SA) and abscisic acid (ABA) levels were determined in both ecotypes in response to S, HL and S + HL (Fig. 4). JA levels significantly increased compared to control values only when Col and Ler plants were subjected to S + HL, whereas the individual stresses did not affect JA content in both ecotypes (Fig. 4a). SA levels decreased in Ler plants subjected to S but increased in response to HL treatment alone or in combination with S, whereas in Col plants, SA levels only decreased with respect to CT in response S + HL (Fig. 4b). ABA levels increased in response to S + HL in both ecotypes, but more prominently in Col plants, whereas individual stresses did not trigger differences in ABA content with respect to control values (Fig. 4c).

### Accumulation of secondary metabolites in Arabidopsis plants subjected to the combination of high salinity and increased irradiance

**Fig. 5:**
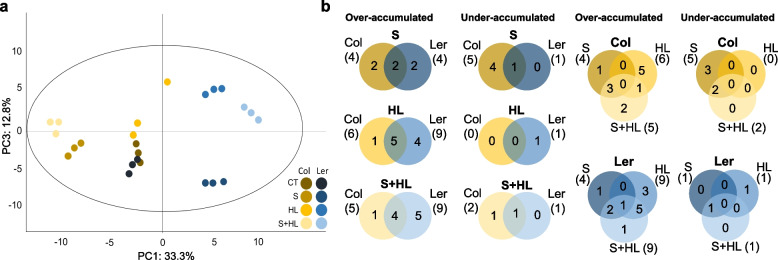
Differential accumulation of secondary metabolites in Col and Ler plants subjected to a combination of high salinity and increased irradiance. **a** Principal Component Analysis (PCA) score plot of metabolite profiles obtained from CT Col and Ler plants, and Col and Ler plants subjected to S, HL and S + HL. **b** Venn diagrams depicting the overlap among over-accumulated and under-accumulated secondary metabolites in Col and Ler plants subjected to S, HL and S + HL. A comparison of the metabolite changes between both ecotypes in each stress condition is shown on the left and a comparison of the metabolite changes among the different stress treatments (S, HL and S + HL) in each ecotype is shown on the right. CT, control; HL, increased irradiance; PCA, principal component analysis; PC, principal component; S, high salinity; S + HL, a combination of high salinity and increased irradiance

**Fig. 6:**
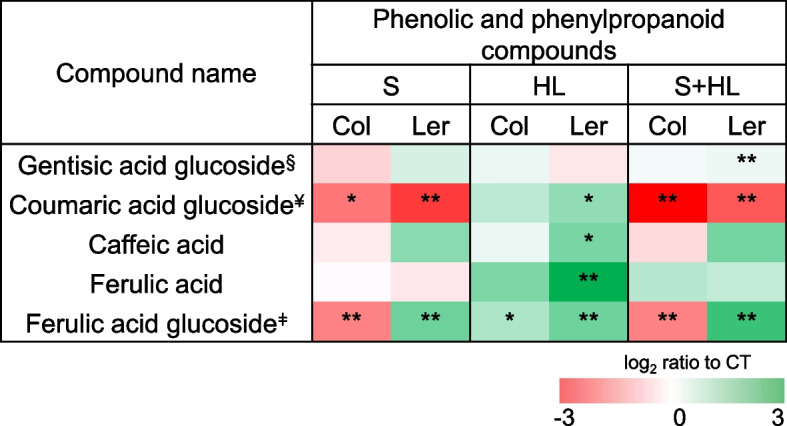
Levels of phenolic and phenylpropanoid compounds in Col and Ler plants subjected to a combination of high salinity and increased irradiance. Levels of phenolic and phenylpropanoid compounds are shown as a heat map in Col and Ler plants subjected to S, HL and S + HL. Metabolite levels are expressed as log_2_ of ratio to control values and are shown as a color scale. ^§^2,5-dihydroxybenzoic acid 5-O-D-glucoside; ^¥^p-Coumaric acid 4-glucoside; ^ǂ^5-Hydroxyferulic acid glucoside. Asterisks denote significant metabolite level (*P < 0.05; **P < 0.01) compared to control conditions. CT, control; HL, increased irradiance; S, high salinity; S + HL, a combination of high salinity and increased irradiance

**Fig. 7:**
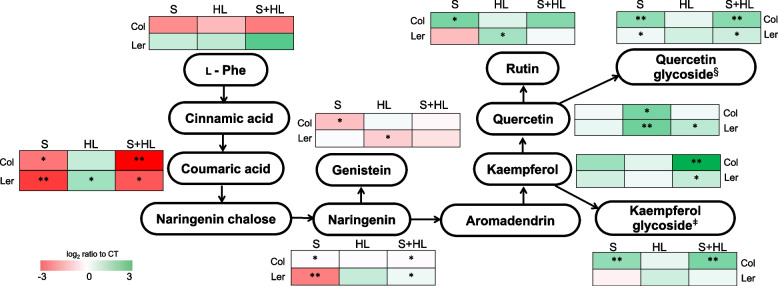
Levels of flavonoids in Col and Ler plants subjected to a combination of high salinity and increased irradiance. Level of flavonoids are shown as heat maps in Col and Ler plants subjected to S, HL and S + HL. Metabolite levels are expressed as log_2_ of ratio to control values and are shown as a color scale. ^§^Quercetin-3-O-(2"-O-rhamnosyl)glucoside-7-O-rhamnoside; ^ǂ^Kaempferol-3-O-glucoside-7-O-rhamnoside. Asterisks denote significant metabolite level (*P < 0.05; **P < 0.01) compared to control conditions. CT, control; HL, increased irradiance; L—Phe, L—phenylalanine; S, high salinity; S + HL, a combination of high salinity and increased irradiance

Differences in the activation of secondary metabolites between Col and Ler plants subjected to high salinity, increased irradiance and the combined factors were analyzed (Figs. 5, 6 and 7; Table 1; Table S3). Principal Component Analysis (PCA; Fig. 5a) plot revealed that, in general, the main source of variation in the data was due to metabolic changes associated with the ecotype, as the first principal component (PC1), explaining a total of 33.3% of total variance, was defined by differences between ecotypes. In turn, principal component three (PC3), explaining a total of 12.8% of total variance, separated the samples based on the metabolic profile of plants subjected to increased irradiance alone or in combination with high salinity (HL and S + HL; Fig. 5a). As shown in Fig. 5b, a different number of secondary metabolites altered under the different stress conditions was observed between Col and Ler. In Col plants, S triggered the over-accumulation of 4 secondary metabolites whereas levels of other 5 decreased under this stress condition. In Ler plants subjected to the same stress condition, increased levels of 4 metabolites were observed whereas only one reduced its concentration compared to CT. S + HL induced the over-accumulation of 5 and 9 secondary metabolites in Col and Ler, respectively, and decreased the content of 9 metabolites in Col and 1 metabolite in Ler. Only 2 and 1 secondary metabolites were found exclusively accumulated in Col and Ler plants subjected to S + HL, respectively (Fig. 5b).

**Table 1:** Identification of secondary compounds analyzed by liquid chromatography coupled with mass spectrometry

Metabolite	m/z	Adduct	Rt (min)
Adenosine	266.0894	M + H^+^	2.573
AMP	346.0549	M + H^+^	1.076
Caffeic acid	179.0330	M + H^+^	5.355
Coumaric acid glucoside	325.0562	M + H^+^	6.023
Ferulic acid	193.0499	M + H^+^	7.098
Ferulic acid glucoside	371.0982	M + H^+^	4.859
Genistein	153.0183	M-H^+^	4.731
Gentisic acid	315.0725	M + H^+^	3.900
Guanosine	282.0842	M + H^+^	2.689
GMP	362.0511	M + H^+^	1.159
Kaempferol	593.1512	M + H^+^	6.675
Kaempferol gly^a^	755.2026	M + H^+^	6.179
Naringenin	579.2068	M + H^+^	7.678
Quercetin	447.0931	M + H^+^	7.585
Quercetin gly^b^	755.2048	M + H^+^	6.386
Rutin	609.1471	M + H^+^	6.146
Uric acid	167.0200	M + H^+^	1.242

*AMP* adenosine monophosphate, *GMP* guanosine monophosphate, *gly* glycoside, *Rt* retention time

^a^Kaempferol-3-O-glucoside-7-O-rhamnoside

^b^Quercetin-3-O-(2"-O-rhamnosyl)glucoside-7-O-rhamnoside

Some of these differentially altered secondary metabolites in response to stress were related to phenolic and phenylpropanoid metabolism (Fig. 6; Table S3). In Ler, levels of gentisic acid glycoside (2,5-dihydroxybenzoic acid 5-O-D-glucoside) and ferulic acid glycoside (5-hydroxyferulic acid glucoside) increased, and coumaric acid glycoside (p-coumaric acid 4-glucoside) content decreased in response to S. In turn, ferulic acid and its glycoside, caffeic acid and coumaric acid glycoside were accumulated in response to HL in Ler. Finally, levels of caffeic acid, ferulic acid glycoside and gentisic acid glycoside increased, and coumaric acid glycoside values decreased in response to S + HL in Ler. In contrast, Col plants showed decreased values of ferulic acid glycoside and coumaric acid glycoside in response to S and S + HL, and increased content of ferulic acid glycoside in response to HL (Fig. 6; Table S3).

In addition to some phenolic compounds and phenylpropanoids, our study identified different flavonoids differentially altered in both wild type ecotypes in response to individual and combined stresses (Fig. 7; Table S3). In Col plants, S induced the accumulation of flavonols including rutin, quercetin glycoside [quercetin-3-O-(2"-O-rhamnosyl)glucoside-7-O-rhamnoside] and kaempferol glycoside (kaempferol-3-O-glucoside-7-O-rhamnoside), whereas levels of some of their precursors such as the flavanone naringenin and the isoflavone genistein, as well as coumaric acid, were under-accumulated compared to CT. In Ler plants, however, this stress did not increase the content of any flavonol but decreased coumaric acid, naringenin and quercetin glycoside contents. HL increased the content of quercetin in both ecotypes, and in Ler plants, also induced an increment in the levels of coumaric acid, rutin and quercetin, as well as a decrease in the content of genistein. Finally, in Col plants subjected to S + HL, coumaric acid diverted into the over-accumulation of different flavonols including kaempferol, kaempferol glycoside and quercetin glycoside. In Ler plants subjected to stress combination, coumaric acid also diverted into quercetin, quercetin glycoside and kaempferol, but to a lesser extent (Fig. 7; Table S3).

### Accumulation of pigments in Arabidopsis plants subjected to the combination of high salinity and increased irradiance

**Fig. 8:**
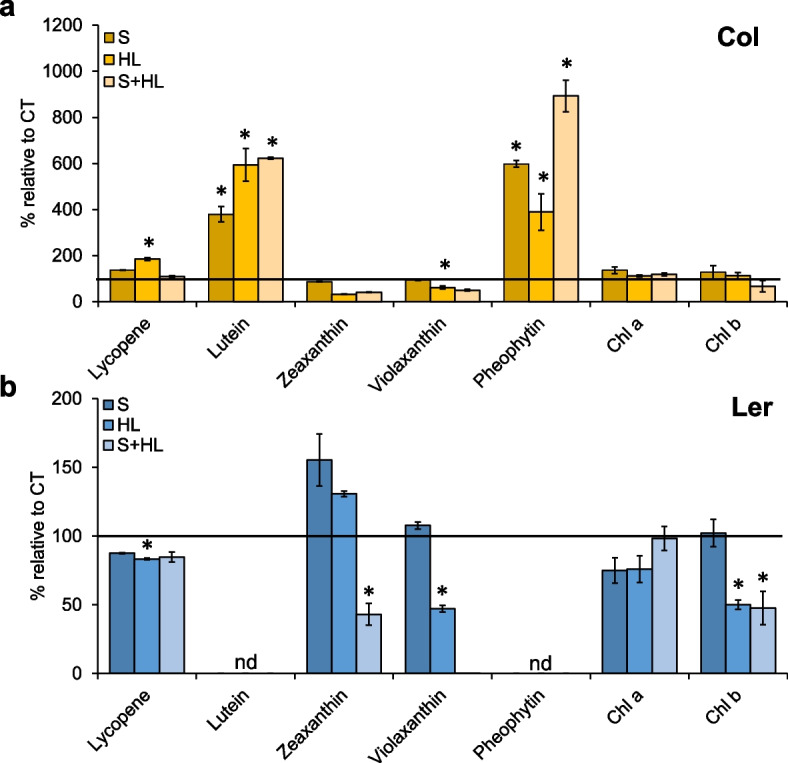
Pigment levels in Col and Ler plants subjected to a combination of high salinity and increased irradiance. **a**, **b** Pigment levels in Col (**a**) and Ler (**b**) plants subjected to S, HL and S + HL. The control levels are represented by a horizontal line set at 100%. Error bars represent SE. Asterisks denote statistical significance at *P* < 0.05 compared to control values. Chl, chlorophyll; CT, control; HL, increased irradiance; n.d., not detected; S, high salinity; S + HL, a combination of high salinity and increased irradiance

Pigment levels were analyzed by liquid chromatographic-mass spectrometry in Col and Ler plants in response to individual and combined stresses. As shown in Fig. 8, different pigments were differentially accumulated between Col and Ler in response to the stress. Lycopene, a carotenoid with antioxidants properties [39, 40], was found over- and under-accumulated in response to HL in Col and Ler plants, respectively, with respect to CT values, whereas both S and S + HL did not alter lycopene levels in both ecotypes. Interestingly, whereas lutein (the most abundant carotenoid in plant photosynthetic tissues; [41]) and pheophytin [42] were over-accumulated in Col plants in response to all stress treatments, these compounds were not detected in Ler plants. Violaxanthin, a xanthophyll pigment, decreased its content in Col plants subjected to S + HL and in Ler plants subjected to HL, whereas the levels of the carotenoid zeaxanthin only decreased its content in Ler plants subjected to S + HL. Stresses did not affect chlorophyll *a* content compared to CT in both wild type plants, but levels of chlorophyll *b* decreased only in Col plants subjected to HL and S + HL (Fig. 8).

## Discussion

In the last decades, several reports have determined the transcriptomic and metabolomic reconfiguration of *Arabidopsis thaliana* plants subjected to different individual or combined abiotic stresses (*e.g*., [13, 15, 43–49]). For example, a transcriptomic study conducted by Rasmussen et al. (2013) [50] revealed how different Arabidopsis ecotypes including Col and Ler responded to individual application of salt, heat and high light stress, highlighting intraspecific transcriptomic variation in responses to these environmental stimuli between both ecotypes. Furthermore, transcriptomic responses to salinity, high light stress and their combination were previously described in Col seedlings [13]. Pathway analysis of transcripts upregulated in response to each stress revealed differences in metabolism regulation at transcriptomic level between the different stresses (Fig. S3; [13]). In addition to transcriptomic studies, some metabolomic studies used different mutants together with their respective wild-type background to show that particular metabolites were key for plant acclimation to specific stress combinations. For example, it was shown that GABA was specifically accumulated in response to the combination of increased irradiance and heat stress and its key role for plant tolerance was demonstrated by using mutants deficient in GABA production (*gad3*) and their wild type plants (Col) [15]. In addition, mutants impaired in ABA biosynthesis and signaling (*aba1-1* and *abi1-1*, respectively), displayed a decreased survival compared to their wild-type background (Ler) plants in response to the combination of drought and heat stress [16], emphasizing the important role of ABA in the tolerance of plants subjected to this stress combination. However, a metabolomic comparison between two of the most used Arabidopsis ecotypes as mutant backgrounds, Col and Ler, in response to the combination of high salinity and increased irradiance has not been assessed yet. Although it was previously shown that both ecotypes differed in their ability to tolerate different abiotic stresses such as drought [33] or high salinity applied in hydroponics medium for 5 days [34], their growth and leaf damage, as well as their quantum yield of PSII were similar in response to the combination of salinity and increased irradiance. However, these similarities were not accompanied by parallel metabolomic responses, and significant differences between both ecotypes were observed in the accumulation of primary metabolites, plant hormones, secondary metabolites as well as some pigments. Similarly, a broad comparison of the metabolic changes triggered by drought and salt acclimation on model and forage legume species of the *Lotus* genus revealed conserved and unique metabolic responses to drought stress, and only few drought-responsive metabolites were conserved among all species tested [51]. In our study, high salinity treatment (S and S + HL) altered the levels of more metabolites than HL, suggesting that S had a major effect on plant metabolism in comparison to HL. Interestingly, the over-accumulation of branched chain amino acids (isoleucine and leucine), as well as proline (known as a major compatible solute in Arabidopsis [52]) observed in both Arabidopsis ecotypes could be a common response of Arabidopsis plants to S + HL and these amino acids could function as compatible osmolytes [53]. In addition, whereas certain amino acids decreased their levels in response to S + HL in Col, other amino acids increased their concentration under these stress conditions in Ler plants. The accumulated amino acids in Ler plants could be as a result of stress-induced protein degradation and these amino acids, especially valine, could function as electron donors in the electron-transfer flavoprotein (ETF)-ubiquinone oxidoreductase complex (ETFQO) to produce ATP in the mitochondria [54, 55]. Further studies are needed to decipher the specific role of these amino acids under the combination of high salinity and increased irradiance.

Sugar accumulation could be a consequence of the stress-related alteration in photosynthesis and can function as a mechanism of stress perception and signaling. Sugars can also work as osmoprotectants and ROS quenchers [56, 57]. Previous reports showed different, and sometimes opposite, patterns of sugar accumulation in plants subjected to different abiotic stress combinations, including Arabidopsis [15, 43, 58, 59], tomato [60], maize [61], or citrus [62]. In our study, high salinity applied individually induced a higher degree of sugar alteration compared to that observed under HL or S + HL in both Arabidopsis ecotypes. In response to S, Ler plants accumulated raffinose and *myo*-inositol (closely related to raffinose biosynthesis; [63]), whereas Col plants accumulated galactinol. Both galactinol and raffinose have been shown to protect plants from oxidative damage due to their role in scavenging hydroxyl radicals under high salinity stress [45], suggesting that Col and Ler could have different strategies to prevent damage caused by salinity-induced oxidative stress. However, other mechanisms could be involved in preventing Arabidopsis plants from oxidative stress under HL and S + HL.

Among other primary metabolites, polyamines are aliphatic compounds accumulated in response to different abiotic stresses that can protect plants from a wide range of different stress-associated damage [64–67]. Despite their demonstrated protective role in plants subjected to individual abiotic stresses, the potential role of polyamines in response to stress combination remains unclear [19]. Our data indicate that, in contrast to Col, Ler plants showed increased levels of ornithine (precursor of citrulline and the polyamine putrescine) in response to S and S + HL, and this increment was parallel to the over-accumulation of citrulline and putrescine. These metabolites were repressed or did not change their content under S and S + HL in the salt-sensitive ecotype Col [68], suggesting that they may play a key role in protecting plants from salinity stress.

In addition to primary metabolites, plant hormones can display specific and unique patterns of accumulation in response to stress combination and their role has been shown to be key for plant acclimation to different combinations of abiotic stressors in many plant species (reviewed in [19]). Our results show a significant increment in JA and ABA levels in both Arabidopsis ecotypes exclusively under S + HL, especially in Col plants. ABA has been proposed to be involved in the acclimation of Arabidopsis plants to heat combined with drought [16] or with high salinity [21], whereas JA could be involved in Arabidopsis tolerance to the combination of high temperatures and increased irradiance [14]. As shown in Fig. 4, S + HL triggered the accumulation of both hormones, and their role could be key for plant tolerance to this stress combination. In addition to ABA and JA, our analysis identified increased levels of SA in response to HL and S + HL only in Ler plants, suggesting that the reduced levels of JA and ABA in Ler compared to Col under S + HL could be compensated by the SA action. Further studies are needed to determine the specific role of plant hormones under this stress combination.

Our work also determined different alterations in secondary compounds in response to S, HL and S + HL in both Col and Ler plants. Despite the low impact that HL had on primary metabolism, its effect on the accumulation of secondary metabolites was similar to that of S and S + HL. Secondary compounds are demonstrated to function in regulating plant defense against different pathogens and herbivores as well as enhance plant acclimation to several environmental stresses such as high salinity, elevated CO_2_, heat, drought or nutrient deficiencies [19, 69–72]. Among the different plant secondary metabolites, phenolic and phenylpropanoid compounds are known to function as potent scavengers of ROS under several abiotic conditions including drought, high salinity, extreme temperatures or heavy metal toxicity [73–75]. Our results showed different patterns of accumulation of phenolic and phenylpropanoid metabolites between Col and Ler plants. Interestingly and in contrast to Col, Ler plants accumulated significant amounts of different phenolic and phenylpropanoid compounds, mainly in response to HL and S + HL, suggesting that Ler plants could display a better antioxidant response under conditions of increased irradiance, applied alone or in combination with high salinity.

Flavonoids are widely shown to accumulate under many abiotic stresses and some of their combinations to act as antioxidant compounds [46, 62, 76–80]. Our data show, in general, similar patterns of flavonoid accumulation between Col and Ler subjected to S + HL. In both ecotypes, S + HL induced flavonol accumulation, mainly kaempferol and quercetin glycoside, whereas the flavanone naringenin and its precursor coumaric acid were under-accumulated in both Col and Ler in response to stress combination. These results suggest that flavonols but not flavanones could have an important role in Arabidopsis responses to stress combination. In contrast, tomato plants subjected to the combination of high salinity and heat decreased flavonol-related compound levels [81], indicating that the accumulation of flavonoids could depend on the plant species as well as the specific stress combination analyzed.

Another interesting finding derived from our metabolic study is the differential accumulation of the carotenoid lutein and the chlorophyll degradation product pheophytin in both Col and Ler ecotypes under the different stresses. Whereas Col accumulated both compounds, they were not detected in Ler plants. Lutein was previously shown to confer photoprotection of PSII under HL or low ultraviolet-B radiation [82, 83], suggesting that this carotenoid could be important for Col to maintain the function of the photosynthetic apparatus under stressful conditions, and that other protective metabolites including phenolic compounds, phenylpropanoids or flavonoids could accomplish this protection in Ler plants. In turn, the accumulation of pheophytin in Col could indicate an enhanced rate of chlorophyll degradation [84], although chlorophyll content did not change with respect to CT in any stress condition. One possible explanation could be an enhanced chlorophyll production in Col plants under stress that results in an increased chlorophyll catabolism as a regulatory mechanism. Further assays are needed to address this intriguing question.

## Conclusions

In summary, the results of this study reveal that both primary and secondary metabolism undergo extensive reprogramming in response to high salinity, increased irradiance and their combination, and that the metabolic alteration under combined stress is unique compared to that of the individual stresses. Although plant performance in terms of growth and quantum yield of PSII was similar between Col and Ler, these ecotypes showed, in general, different mechanisms to tolerate the combination of an acute and short-term stress (high irradiance), and a long-term stress (increased salinity). These stress-responsive strategies included relocation of amino acids and sugars to act as potential osmoprotectants, and the accumulation of different stress-protective compounds including polyamines as well as phenolic compounds, phenylpropanoids and flavonoids. Of course, further studies are required to explore the effects and responses of plants subjected to chronic application of both stresses. Our analysis should be viewed, therefore, as an initial identification of different pathways and metabolites that differentially change under S + HL and that could be considered in genetic approaches of (combined) abiotic stress response using Arabidopsis mutants whose genetic backgrounds are Col or Ler.

## Methods

### Plant material and growth conditions

*Arabidopsis thaliana* Columbia-0 (Col) and Landsberg erecta-0 (Ler) plants were grown in peat pellets (Jiffy7; http://www.jiffygroup.com/) at 23 °C/18 °C light/dark temperatures under long-day growth conditions (16-h light from 7 AM to 11 PM [cool-white fluorescent bulbs]; 75 μmol m ^−2^ s^−1^ /8-h dark from 11 PM to 7 AM; Fig. S1; see light spectrum in Fig. S2).

### Stress treatments

Three different stress treatments were performed in parallel: individual high salinity (S), individual increased irradiance (HL), and combined conditions of high salinity and increased irradiance (S + HL) (Fig. S1). To prevent lethality that could potentially result from conditions of stress combination, the intensity and duration of each of the individual stresses were calibrated based on our previous studies to ensure minimal impact on plant growth and survival [14, 21]. Long-term S treatment was applied by watering 15-day old plants with a water solution containing 150 mM NaCl for ten days. Short-term HL was applied by exposing 25-day old plants to 650 μmol m^−2^ s^−1^ (Hortimax agro, Cmh; Vanguard; see light spectrum in Fig. S2) for 7 h (from 8 AM to 3 PM) at the end of the experimental period. The combination of S and HL (S + HL) was performed by simultaneously subjecting plants to 650 μmol m^−2^ s^−1^ light for 7 h (from 8 AM to 3 PM) and watering with a water solution containing 150 mM NaCl for ten days as shown in Fig. S1. Control (CT) plants were maintained under control growth conditions as explained above. At the end of the stress treatments, percentage of damaged leaves and rosette diameter were recorded in each experimental group of plants. Light intensity was measured by using a quantum photo-radiometer data logger (DO9721, Delta OHM, Italy). All experiments were repeated three times. For each independent experiment, leaves from 30 plants per ecotype and stress treatment were pooled and considered as a biological replicate.

### Quantum yield of photosystem II

The quantum yield of PSII (Φ_PSII_; [85, 86]) was measured using a portable fluorometer (FluorPen FP 110/S, Photon Systems Instruments, Czech Republic) on illuminated leaves, at the end of the stress period. At least two fully expanded, young leaves from three plants were used per ecotype and stress treatment.

### RT-qPCR analysis

Relative expression analysis by RT-qPCR was performed according to [87]. Approximately 50–75 mg of rosette leaf tissue was used to isolate total RNA using the RNeasy Mini Kit from Qiagen, following the manufacturer's instructions. To determine the concentration of total RNA, spectrophotometric analysis with Nano-Drop (Thermo Scientific, Wilmington, DE, USA) was employed, and the RNA purity was measured by the ratio of absorbance readings at 260 nm and 280 nm. Primescript RT reagent (Takara Bio, Inc. Japan) was employed for reverse transcription, and 1 μg of total RNA was used. The subsequent RT-qPCR analysis was carried out on a StepOne Real-Time PCR system (Applied Biosystems, CA, USA). The reaction involved 5 μL of SYBR Green (Applied Biosystems), 1 μL of cDNA, and 1 μM of each gene-specific primers (tubuline AT5G62690: F—CAACTCTGACCTCCGAAAGC, R—CTTGGAGTCCCACATTTGCT; Zat12 AT5G59820: F – TGGGAAGAGAGTGGCTTGTT, R – TAAACTGTTCTTCCAAGCTCCA). Three technical repeats were analyzed on each biological replicate.

### Malondialdehyde analysis

To isolate the MDA content, approximately 200 mg of frozen leaf tissue was used, which was ground and homogenized in 2 mL of 80% ethanol (Panreac) using sonication (30 min). After homogenization, the samples were centrifuged at 12000 g for 10 min. The resulting supernatant was then divided into separate portions. A portion was combined with 20% trichloroacetic acid and another portion with a combination of 0.5% thiobarbituric acid and 20% trichloroacetic acid. These combinations were incubated at 90 °C for 1 h in a water bath. Following incubation, the samples were allowed to cool, and then centrifuged at 5000 g for 5 min at 4 °C. The absorbance of the resulting supernatant was measured at 440 nm, 534 nm, and 600 nm. The calculation of MDA concentration was performed as explained in [88].

### Metabolite extraction

Metabolite extraction was performed according to [89] using 50 mg of pulverized freeze-dry leaves from three independent biological repeats for each stress condition and ecotype. Three technical repetitions were performed for each biological replicate. Metabolites were extracted using a mixture of 1 mL of methyl-*tert*-butyl-ether:methanol (3:1) along with 0.3 μg mL^−1^ isovitexin, 0.5 μg mL^−1^ of ribitol, and phosphatidylcholine as internal standards for secondary metabolites, primary metabolites and pigments, respectively. The samples were subjected to 10 min of agitation on an orbital shaker at 4 °C, followed by 10 min of sonication in an ice-cooled bath. Afterward, 0.5 mL of a H_2_O:MeOH (3:1 v/v) solution was added, and samples were vortexed. Next, the samples were centrifuged for 5 min at 10,000 g at 4 °C to fractionate the metabolites by phase separation. The polar and non-polar fractions were aliquoted and both fractions were then concentrated using a speed-vac. The resulting dry pellets were stored at -80 °C until they were needed for further use. Three independent extractions were performed per biological replicate.

### Determination of primary metabolites

For primary metabolite analysis, speed-vac dry pellets from 130 μL of the polar fraction were derivatized with 40 μL of 20 mg mL^−1^ methoxyamine hydrochloride in pyridine at 37 °C for 120 min in agitation. Subsequently, 70 μL of N-Methyl-N-trimethylsilyltrifluoroacetamide (MSTFA) was added, and samples were incubated shaking at 37 °C for 30 min [90]. To inject the samples into a gas chromatograph coupled to a time-of-flight mass spectrometer (GC–MS) (Leco Pegasus HT TOF–MS; LECO Corporation, St. Joseph, MI, USA), an autosampler Gerstel Multi-Purpose system (Gerstel GmbH & Co.KG, Mülheim an der Ruhr, Germany) was used. Chromatography and mass spectrometry conditions were exactly as described in [90]. Mass chromatograms were assessed with Chroma TOF 4.5 (Leco) and TagFinder 4.2 software. Metabolites were identified by comparing their mass spectra and retention indices with those of standards. Peak areas in each chromatogram were then adjusted to the internal standard (ribitol) area, and also to the exact sample weight.

### Determination of secondary metabolites

For secondary metabolite analysis, dry pellets from 260 μL of the polar fraction were resuspended in 400 μL of 50% MeOH by vortexing. Resuspended samples were sonicated for 3 min followed by centrifugation at 4000 g for 5 min. Subsequently, samples were injected in a UPLC-MS equipped with an HSS T3 C18 reverse-phase column (100 × 2.1 mm internal diameter, 1.8 μm particle size; Waters) at 40 °C. The mobile phase was composed of two components: 0.1% formic acid in water (solvent A), and 0.1% formic acid in acetonitrile (solvent B). Chromatographic conditions and mass spectrometry were exactly as described in [91]. Processing of chromatograms, peak detection, and integration were performed using RefinerMS (version 5.3; GeneData). Metabolite identification and annotation were performed as reporting standards [92]. On using our in-house reference compounds library, 10 ppm mass error, and a dynamic retention-time shift of 0.1 were allowed [92].

### Determination of pigments

For pigments analysis, 450 μL of the non-polar fraction were resuspended in 200 μL of an acetonitrile:isopropanol (7:3) solution by vortexing. Samples were sonicated for 5 min and centrifuged for 2 min at 10,000* g*. Then, 90 μL of the supernatants was used to inject it in a UPLC-MS system. Chromatographic conditions and mass spectrometry were exactly as described as described in [93]. Briefly, samples were injected on a C18 reversed phase column (100 × 2.1 mm internal diameter; 1.8 μm particle size; Waters). As mobile phases, 0.1% acetic acid in water (solvent A) and 0.1% acetic acid in acetonitrile:isopropanol (7:3) (solvent B) at 400 μL min^−1^ of flow rate were used. Mass spectra were assessed from min 0 to 19 of the UPLC gradient, covering a 100–1500 m/z of mass range. Molecular masses, retention times and associated peak intensities were extracted from raw files by using RefinerMS v5.3 from GeneData, and Xcalibur from Thermo Fisher Scientific as described in [93]. Pigments were identified, annotated and quantified as described in [93].

### Hormone profiling

Hormone extraction and analysis were carried out as described in [94] with a few modifications. Six technical replicates were performed per biological replicate. Briefly, a mixture consisting of 50 ng of [C_13_]-SA, [^2^H_6_]-ABA, and dihydrojasmonic acid (DHJA) was added to 50 mg of frozen leaf tissue. Then 1 mL of cold acetonitrile:water solution (1:1) was added and the mixture was homogenized in a ball mill (MillMix20; Domel). Following ultrasonication for 5 min, a centrifugation at 4000* g* and 4 °C was performed, and supernatants were recovered. Before hormone extraction, HLB 1 cc cartridges (Oasis SPE; Waters) were activated by adding 1 mL of ultrapure methanol followed by 1 mL of ultrapure water. To equilibrate the column, 1 mL of cold acetonitrile:water solution (1:1) was added. Supernatants were then injected into individual columns and the flow was discarded. Subsequently, 0.3 mL of cold acetonitrile:water (3:7) solution was added to the column to elute the extracts. Extracts were then transferred to HPLC vials and injected into an ultra-performance LC system (Acquity SDS; Waters). Chromatographic separations were performed on a reversed-phase C18 column (Gravity, 50 × 2.1 mm, 1.8-μm particle size; Macherey–Nagel) using an acetonitrile:water (supplemented with 0.1% [v/v] formic acid) gradient (flow rate was 300 mL min^−1^) as detailed in [95]. Hormones were quantified with a TQ-S Triple Quadrupole Mass Spectrometer (Micromass).

### Statistical analysis

Metabolite data were represented as ratio to control and were statistically analyzed by using MetaboAnalyst [96]. Three biological replicates were considered for all the statistical analyses, each corresponding to the average of three technical replicates (six in the case of hormones). * means *P* < 0.05 and ** means *P* < 0.01. Data for hormone profiling, rosette diameter and quantum yield of PSII were represented as mean of three biological replicates ± SE. Statistical analysis were performed by two-way ANOVA followed by a Tukey post hoc test (*P* < 0.05) when a significant difference was detected (different letters denote statistical significance at *P* < 0.05) or by two-tailed Student’s t-test for RT-qPCR analysis (* means statistical significance at *P* < 0.05 with respect to control).

### Supplementary Information

**Additional file 1:** **Fig. S1. **The experimental design used for the metabolomic study of high salinity (S), high irradiance (HL), and a combination of high salinity and high irradiance (S+HL) using Arabidopsis Col and Ler plants. CT, control; HL, high irradiance; S, high salinity; S+HL, a combination of high salinity and high irradiance. **Fig. S2. **Light spectrum used for growing plants under control conditions and during increased irradiance treatments. CT, control; HL, increased irradiance. **Fig. S3. **Pathway analysis of transcripts upregulated in response to S, HL, and S+HL using Arabidopsis Col seedlings. Transcriptomic data was obtained from [13]. Numbers in bars depict *p*-value. HL, increased irradiance; S, high salinity; S+HL, a combination of high salinity and increased irradiance.

**Additional file 2:** **Supplementary Table 1.** Levels of primary metabolites in Col and Ler plants subjected to high salinity (S), increased irradiance (HL), and the combination of S and HL (S+HL). Metabolite levels are expressed as the fold change compared to control conditions. **Supplementary Table 2.** Levels of metabolites involved in glutamate metabolism in Col and Ler plants subjected to high salinity (S), increased irradiance (HL), and the combination of S and HL (S+HL). Metabolite levels are expressed as the fold change compared to control conditions. Abbreviations used: CT, control; GABA, γ-aminobutyric acid; HL, increased irradiance; S, high salinity; S+HL, a combination of high salinity and increased irradiance; SE, standard error. **Supplementary Table 3.** Levels of secondary metabolites in Col and Ler plants subjected to high salinity (S), increased irradiance (HL), and the combination of S and HL (S+HL). Metabolite levels are expressed as the fold change compared to control conditions. Abbreviations used: CT, control; HL, increased irradiance; S, high salinity; S+HL, a combination of high salinity and increased irradiance; SE, standard error.

## Data Availability

The datasets supporting the conclusions of this article are included within the article and its Supplementary Figures and Tables.

## Abbreviations

ABA: Abscisic acid
Chl: Chlorophyll
CT: Control
GABA: γ-Aminobutyric acid
HL: Increased irradiance
JA: Jasmonic acid
S: High salinity
SA: Salicylic acid
S + HL: The combination of high salinity and increased irradiance
