# Supplementary material for: Comparison of metabolomic reconfiguration between Columbia and Landsberg ecotypes subjected to the combination of high salinity and increased irradiance
Source: BMC Plant Biol. 2023 Aug 25;23:406. doi: 10.1186/s12870-023-04404-7 (PMC10463500; doi:10.1186/s12870-023-04404-7)
Supplement: Supplementary file 1 — Additional file 1: Fig. S1. The experimental design used for the metabolomic study of high salinity (S), high irradiance (HL), and a combination of high salinity and high irradiance (S+HL) using Arabidopsis Col and Ler plants. CT, control; HL, high irradiance; S, high salinity; S+HL, a combination of high salinity and high irradiance. Fig. S2. Light spectrum used for growing plants under control conditions and during increased irradiance treatments. CT, control; HL, increased irradiance. Fig. S3. Pathway analysis of transcripts upregulated in response to S, HL, and S+HL using Arabidopsis Col seedlings. Transcriptomic data was obtained from [13]. Numbers in bars depict p-value. HL, increased irradiance; S, high salinity; S+HL, a combination of high salinity and increased irradiance. [file 12870_2023_4404_MOESM1_ESM.pdf]

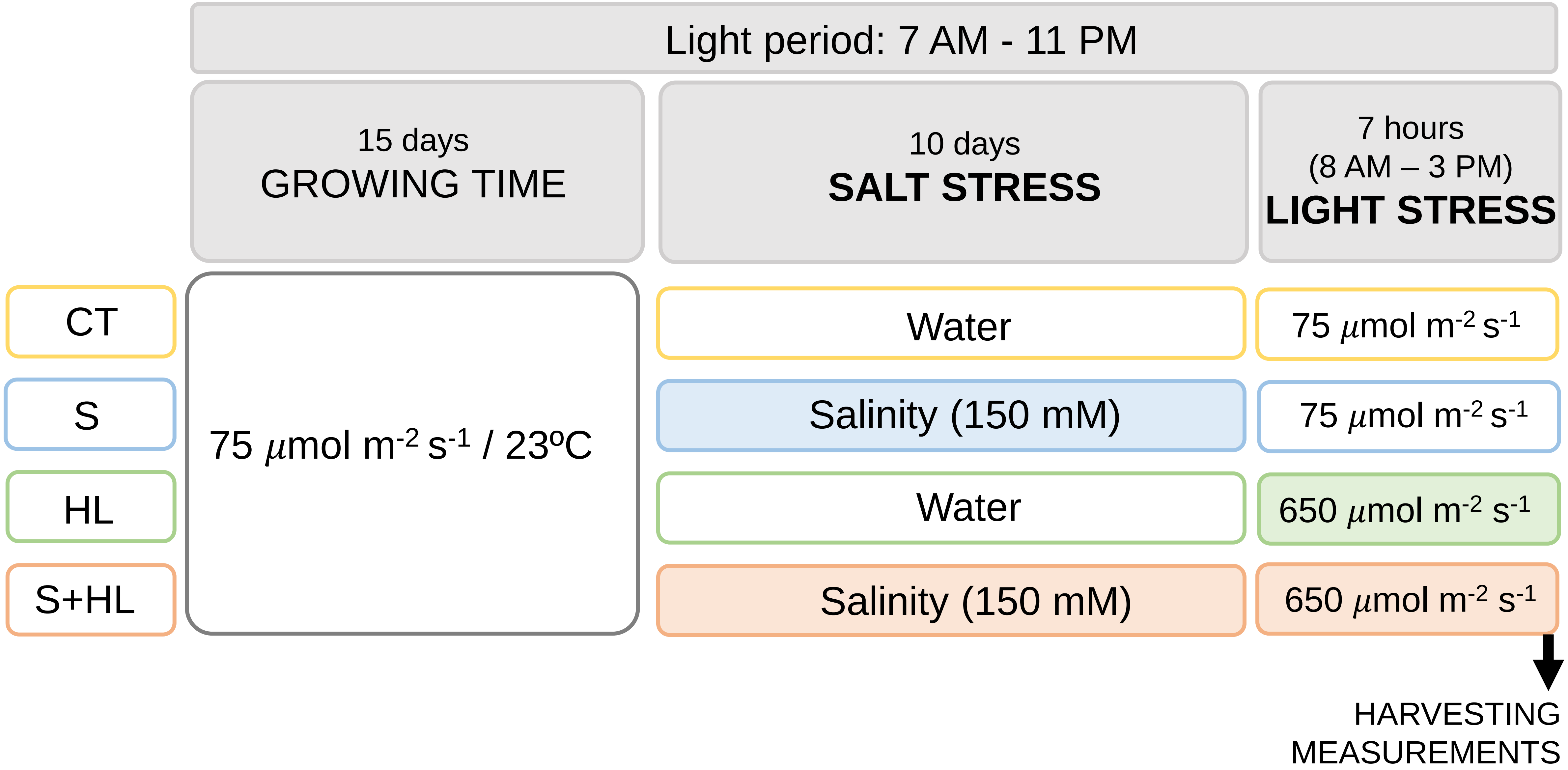

Fig. S1. The experimental design used for the metabolomic study of high salinity (S), high irradiance (HL), and a combination of high salinity and high irradiance (S+HL) using Arabidopsis Col and Ler plants. CT, control; HL, high irradiance; S, high salinity; S+HL, a combination of high salinity and high irradiance.

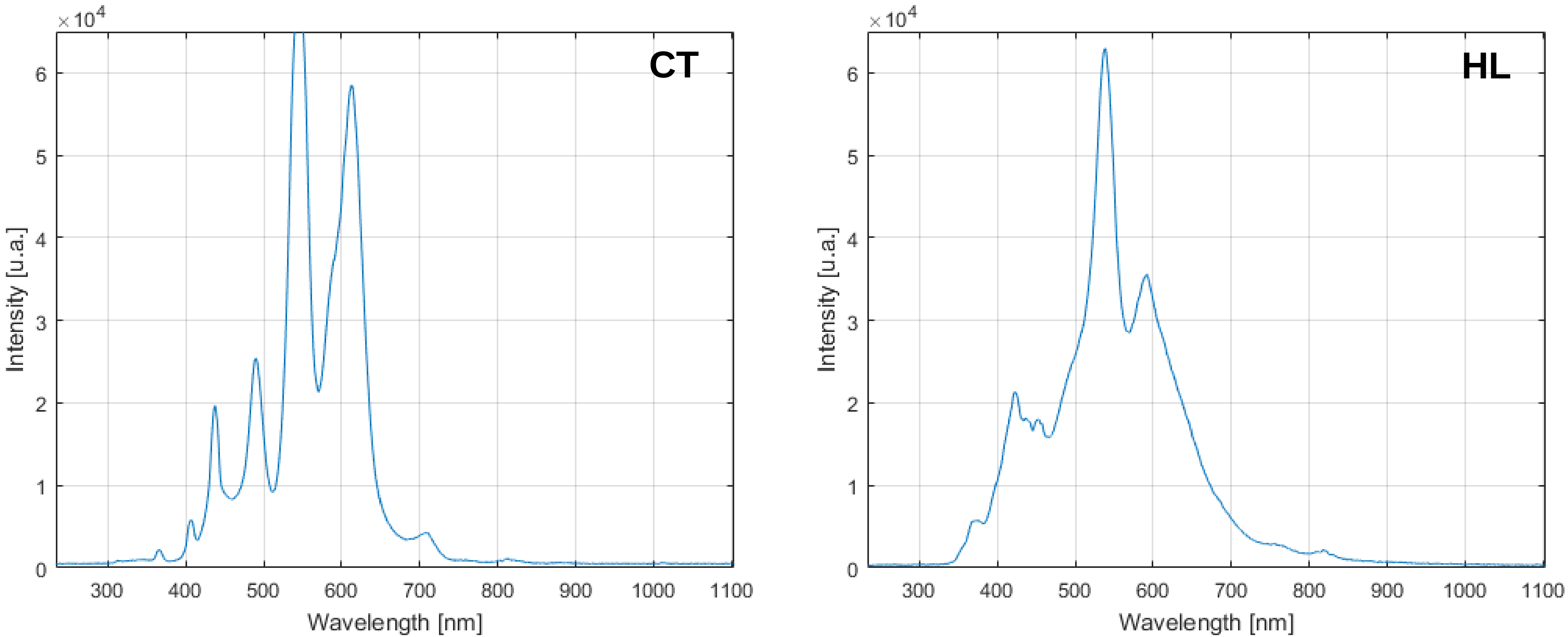

Fig. S2. Light spectrum used for growing plants under control conditions and during increased irradiance treatments. CT, control; HL, increased irradiance.

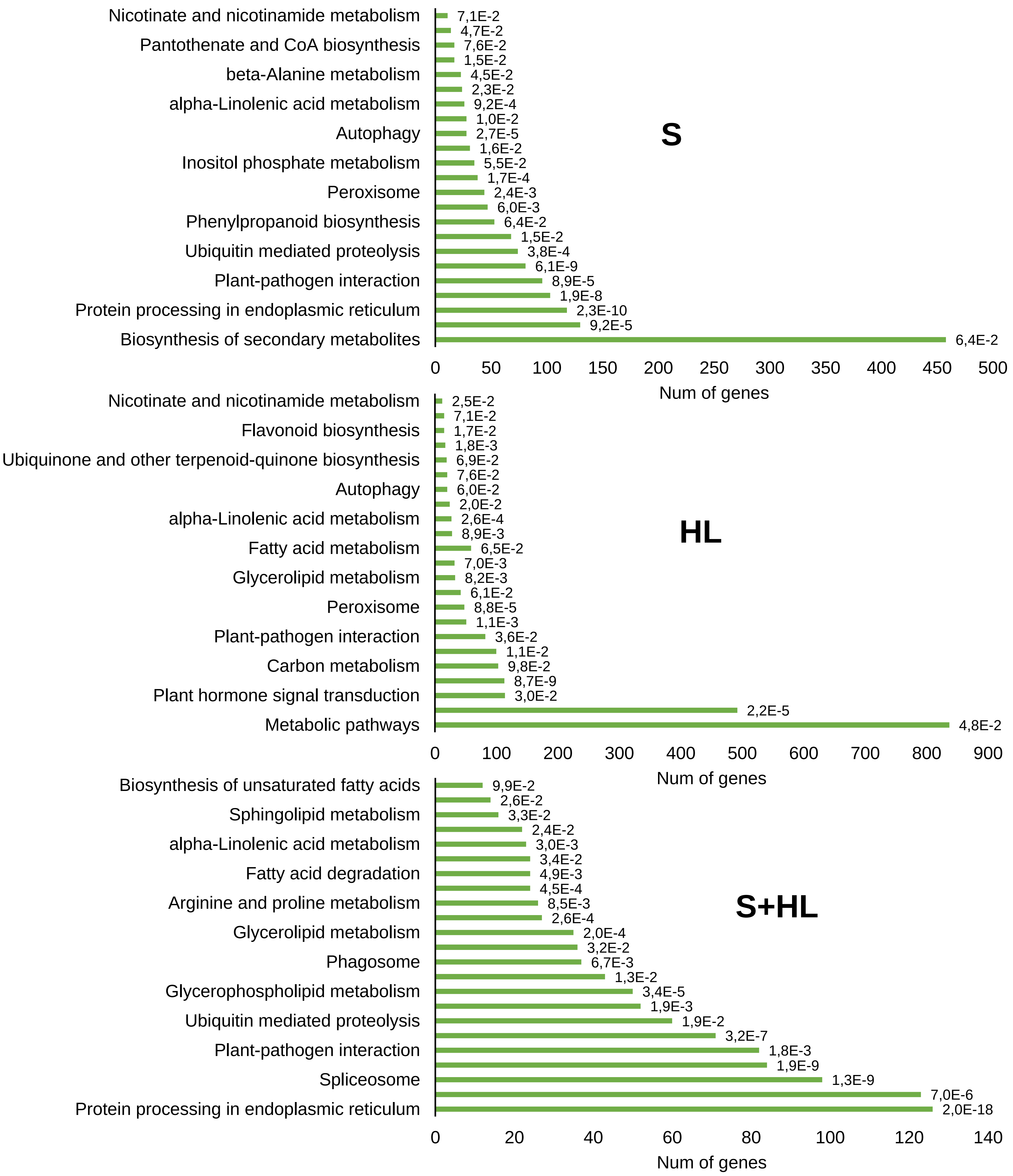

Fig. S3. Pathway analysis of transcripts upregulated in response to S, HL, and S+HL using Arabidopsis Col seedlings. Transcriptomic data was obtained from [13]. Numbers in bars depict p-value. HL, increased irradiance; S, high salinity; S+HL, a combination of high salinity and increased irradiance.
